# Hydraulic Fracturing: Paving the Way for a Sustainable Future?

**DOI:** 10.1155/2014/656824

**Published:** 2014-03-25

**Authors:** Jiangang Chen, Mohammed H. Al-Wadei, Rebekah C. M. Kennedy, Paul D. Terry

**Affiliations:** Department of Public Health, 390 HPER Building, 1914 Andy Holt Avenue, University of Tennessee, Knoxville, TN 37996, USA

## Abstract

With the introduction of hydraulic fracturing technology, the United States has become the largest natural gas producer in the world with a substantial portion of the production coming from shale plays. In this review, we examined current hydraulic fracturing literature including associated wastewater management on quantity and quality of groundwater. We conclude that proper documentation/reporting systems for wastewater discharge and spills need to be enforced at the federal, state, and industrial level. Furthermore, Underground Injection Control (UIC) requirements under SDWA should be extended to hydraulic fracturing operations regardless if diesel fuel is used as a fracturing fluid or not. One of the biggest barriers that hinder the advancement of our knowledge on the hydraulic fracturing process is the lack of transparency of chemicals used in the practice. Federal laws mandating hydraulic companies to disclose fracturing fluid composition and concentration not only to federal and state regulatory agencies but also to health care professionals would encourage this practice. The full disclosure of fracturing chemicals will allow future research to fill knowledge gaps for a better understanding of the impacts of hydraulic fracturing on human health and the environment.

## 1. Introduction

The United States struggles with increasing carbon emissions due to the use of high-carbon energy sources such as petroleum and coal, which together provide the largest portion of primary energy consumption in the country [1]. Energy-related activities have been the primary source of domestic anthropogenic greenhouse gas (GHG) emissions which contributes to the widespread climate-related stress on water resources, livestock, ecosystems, and human health [2]. This appreciation therefore highlights the link between our primary future energy source(s) and future climate change and impacts.

Solar, wind, biomass waste, and geothermal and hydroelectric energy have long been recognized as renewable and sustainable energy resources; currently however, they only comprise 9% of our energy consumption; this is in sharp contrast to the rapid growth of national natural gas market production with a record high of 25,319 billion cubic feet (717 billion cubic meters) in 2012 [3]. In fact, natural gas contributed approximately 27% of the total United States energy consumption and accounted for 40% of industrial and 74% of commercial and residential energy consumption in 2012 [1, 3]. Although the accuracy of GHG emission estimates from natural gas production and usage is still a matter of debate [4, 5], natural gas, which is composed mainly of methane, is considered cleaner-burning than coal or oil with significantly lower levels of carbon dioxide, nitrogen oxides (NOx), sulfur dioxide, and particles emission when combusted.

The United States is the largest natural gas producer in the world [6]. Total marketed production grew by 7.9 percent in 2011, which was the sixth consecutive year of growth in marketed production and the largest year-over-year percentage increase since 1984. The increase in natural gas production in United States came exclusively from the onshore which was largely concentrated in shale plays [7]. Largely located in western, midwestern, and northeastern areas of the country, shale gas accounts for about 25% of the total domestic natural gas production with its contribution is still rapidly growing [8]. The utilization of shale gas as a resource would not be considered practical and commercially profitable without the emergence of hydraulic fracturing.

Hydraulic fracturing is an advanced stimulation technological process accompanied by increasing lateral horizontal well drilling (more than 1 km or 3000 ft) and the injection of fracturing fluid under high pressure (480–850 bar) to open new or enlarge the existing rock fractures that facilitate the migration of natural gas toward the surface. Hydraulic fracturing could create a contact area that is thousands of times greater than that achieved by the typical method of vertical drilling therefore significantly increases the production of natural gas from a single well site [9].

Hydraulic fracturing fluid is essential for creating fractures. Fracturing is conducted in multiple stages, and wells may be refractured multiple times to maximize its economic life [8]. Based on formation characteristics, different combinations of fracturing fluids might be used to enhance fracturing effectiveness. While oil, water, methanol, or a combination of water and methanol are used as fracturing fluids in practice, the predominant types of fracturing fluids for unconventional shale gas extractions are water based and this review therefore will focus on the overall impact of water based hydraulic fracturing activities on surface and groundwater.

The fracturing fluid is comprised of approximately 90% water to effectively create thin, long fractures distributed over a larger area, 9% sand (natural occurring sand grains, resin coated sand, high-strength ceramic materials, and resin coated ceramic materials) which serves as proppant to hold or prop open the fractures, and a small percentage of additives serving various purposes [10]. Not all of the additives are used in every hydraulically fractured well (see [11] for a list of additives used in hydraulic fracturing). The composition and proportion of fracturing fluid chemistry is designed according to the geological characteristics of each target formation. As part of the fracturing fluid, acids are used to clean wellbore and dissolve near-wellbore acid soluble minerals (i.e., Calcite CaCO_3_) to create open conduit for the hydraulic fracturing fluid. For example, hydrochloric acid is one of the most commonly used acids with a typical concentration range of 0.08%–2.1% of the total fluid pumped (as volume of 15% HCl) [12, 13]. Due to the corrosive nature of acids, a corrosion inhibitor at a concentration of 2,000 to 5,000 ppm which equates to 0.0004%–0.0043% of the total fluid volume is added into the fracturing fluid. The acid inhibitor bonds to the surface of metal minimizing the galvanic corrosion of steel tubes, well casing, tools, and fracturing fluid containment tanks, to prevent potential chemical leakage [12, 13]. Higher concentrations of the corrosion inhibitor are required when casing and tubing have a higher composition of metal or when downhole temperatures exceed 250 degrees Fahrenheit (121°C) [13, 14].

To function properly, fracturing fluids need to be viscous enough to create a fracture of adequate width while at the same time be able to travel further away from the well pad to extend fracture length before settling [15]. To achieve this, a more viscous fluid, lighter proppant, or a combination can be applied. There are usually two ways to increase a fluid viscosity. One way is to add a gelling agent, a polymer such as guar or guar derivatives. However, in the presence of high temperatures, the gelled fluid could lose its viscosity, a problem that could be resolved by increasing the polymer concentration or adding cross-linking agents to increase the molecular weight of the solution [14–16]. While the addition of these agents could further increase viscosities by several orders of magnitude, these relative large agents can plug the small pores of the fracture surface and decrease the gas flow [15]. Alternatively, a foam-based fracturing fluid could be used which gives an effective viscosity similar to that of a gelled fluid. The addition of foam in fracturing fluid has the advantage of minimizing the water use, reducing the leaking-off rate, and improving fluid recovery efficiency, although the tradeoffs could be high cost, high surface pump pressure, and low proppant load [15].

Practically speaking, fracturing fluids also include potassium chloride, breakers, biocides, fluid-loss additives, and friction reducers [12, 14, 15]. While most companies rely on the use of gel additives to increase fracture fluid viscosity, in practice, others also add small amounts of potassium chloride to further enhance fracturing fluids viscosity, a step that could reduce the amount of gel required to achieve the same level of viscosity [15]. This approach is considered by some companies to be environmentally “responsible” or “safe” because the current environmental impact of gels is still largely unknown and potassium chloride is a nonapparent health hazard at low concentration [10, 12, 16]. In addition to increasing the fracturing fluid viscosity, 1–3% potassium chloride solutions have been applied in formations to stabilize the clay and prevent its swelling due to the presence of water [17].

Biocides, such as glutaraldehyde, are added to eliminate bacterial growth in fracturing fluids [12, 14, 16]. The growth of bacteria in the presence of organic materials in fracturing fluid can produce corrosive byproducts and enzymes that interfere with gel formation and thereby reduce fluid viscosity [17]. Friction reducers, which include latex polymers or copolymers of acrylamides, are generally mixed with the fracturing fluid at a concentration range of 0.25 to 2.0 pounds per 1,000 gallons (0.11 to 0.90 kg per 3,785 liters) of fluid to minimize the loss of pressure due to the presence of friction between fracturing fluid and tubing/wellbore during the fracturing [12, 16]. Breakers, such as ammonium persulfate, are used in fracturing fluid, to allow a delayed breakdown of the “crosslinker” and “gel” in the formation to reduce fracturing fluid viscosity thereby enhancing postfracturing fluid recovery and flowback [12, 16].

Currently, there are no federal disclosure standards mandating hydraulic fracturing companies to disclose a list of their toxic chemicals [18]. As shown in Table 1, of the 29 states with hydraulic fracturing activity ongoing, only 15 states enact disclosure laws. Of these 15 states, nine states are exempted from disclosing chemicals that serve as trade secrets, and only one of these 9 states (Wyoming) has a process in place for the state evaluation of trade secrets with a factual justification required. Six of the 15 states, however, require the disclosure of trade secret chemicals to health care professionals under emergency circumstances for effective patient treatments but four of the six states further require physicians to sign confidentiality contracts to receive such disclosure which potentially may slow the flow of information during emergencies [18]. Without full disclosure of all chemicals used in fracturing process, it will be difficult to assess the health and environmental impacts [19, 20].

## 2. Impact of Hydraulic Fracturing on Water

Before the emergence of new advanced technology, hydraulic fracturing will continuously play an essential role to facilitate the expansion of natural gas development [21]. Two decades ago, available scientific research was focused more on how to improve the efficiency of hydraulic fracturing performance rather than its environmental impacts. Hydraulic fracturing is performed in several stages of varying distance to stimulate the entire length of the well [22]. Each stage requires tens of thousands of barrels of water which can total up to several million gallons per well [22]. The scarcity of peer-reviewed data addressing any association between the technology and the availability and quality of local water resources has been identified as the foremost issue among other social, economic, and environmental concerns [23].

### 2.1. Large Volume of Water Withdraw for Fracturing Activity

Hydraulic fracturing requires the use of large volumes of water for a single operation. Fracturing shale gas typically requires the withdrawal of 2.3–3.8 million gallons (8.7–14.4 million liters) of water per single well [23, 24]. Recent data, however, indicate that the volume of water used during hydraulic fracturing may be underestimated [25]. The amount of water required for fracturing treatments therefore could vary depending on the type of well drilled and its geological location. In general, the deeper the well is and the stronger the rock formation is, the more water is needed for the fracturing process [21, 26].

Industry argues that water used in the hydraulic fracturing process is insignificant compared to the total annual water withdrawal in the United States. In 2005, approximately 149,650 billion gallons (1,552 billion liters per day) of water was withdrawn for various uses in the United States [27]. The largest two sectors for water withdrawal in 2005 were used for thermoelectric power generation (49%) and irrigation (31%) [27]. In contrast, less than 1.0% of water withdrawal was used for mining purposes which includes water used for extracting solid minerals, such as copper; liquids, such as petroleum; and gases, such as natural gas [27]. Even though the booming of hydraulic fracturing process across the nation in most recent years could imply more water withdrawal compared to 2005, the portion of the water withdrawal overall used for natural gas production after 2005 should not change significantly [27].

Nonetheless, even 2 million gallons (7.6 million liters) of water used per well could be significant, simply because the water is usually taken directly from one single location and in many cases from remote and environmentally sensitive areas. While total hydraulic fracturing water use represents less than 1.0% of the water use in the nation [27], the hydraulic fracturing water use is unevenly distributed across individual states and may locally represent a higher fraction of the total water use which can result in a significant impact on local flow regime [28, 29]. Texas for instance, is an area with a wet and dry season. Total water use for hydraulic fracturing (for oil and natural gas) in Texas has increased by about 125%, from 36,000 acre feet (AF) (0.04 cubic kilometers) in 2008 to about 81,500 AF (0.1 cubic kilometers) in 2011 [30]. During the dry seasons, the withdrawal of large volumes of water for fracturing processes could significantly limit water availability for human consumption, crop, and livestock use [23]. Furthermore, since hydraulic fracturing has expanded to the drier southern and western parts of the state of Texas, the industry might have to adapt to those new conditions by reducing fresh water consumption and increasing water recycling and reuse [30].

### 2.2. Hydraulic Fracturing and Local Water Quality

Once hydraulic fracturing is complete, the pressure in the well is released by the removal of the pressure barriers such as the frac plugs [31]. Typically, two types of waste fluids, the flowback fluid and produced fluid, will be brought back to the surface from hydraulically fractured wells. The completion of hydraulic fracturing is accompanied with the quick flow back of hydraulic fluid mixed with brine (termed as “flowback” water) from the formation to the surface right before the well is placed into production for an average period of two to four weeks [10, 32]. The flowback will relieve the downhole pressure and allow gas migration to the surface. Once the well is placed into production, waste fluid (termed as “produced water” which includes subsequently returned hydraulic fluids and natural formation water) is continuously coproduced with gas over the lifetime of the well [33]. The amount and composition of wastewater generated by a particular well varies, however, greatly depending upon the geologic formation from which it originates, the extraction method utilized in the natural gas production process, and the chemicals (i.e., corrosion inhibitors and breakers) selected for the process [33–35]. The waste fluid (flowback as well as produced water) contains brine, fracturing fluid additives, hydrocarbons, and suspended and dissolved constituents from the shale formation and sometimes naturally occurring radioactive materials [10, 32–34]. The longer the fluid takes to return to the surface, the greater the concentration of formation materials will be found in the waste fluid [10, 36]. The water fluid is usually stored in onsite tanks or pits and is later treated either onsite or in another facility during the waste management process to reduce the toxicity of the fluid and minimize its environmental impacts.

Between 10 and 80% of the injected fracturing fluid volume may return to the surface as wastewater [36]. Both inorganic and organic constituents exist in flowback and produced water with in general inorganic components being much more extensive and prevalent than those of organic constituents [37]. The waste fluid generated from hydraulic fracturing wells is managed by deep well disposal, onsite treatment, reuse, or transportation offsite to treatment facilities followed by surface discharge [37]. The Pennsylvania Department of Environmental Protection (PADEP) requested that unconventional natural gas drillers voluntarily stop sending the wastewaters to Publicly-Owned Treatment Works (POTW) or Private Centralized Wastewater Treatment (CWT) within the commonwealth by May 19, 2011 [38], both of which might not be equipped to handle hydraulic fracturing wastewater [36]. In POTW of Pennsylvania, a typical treatment process includes a pretreatment to remove total suspended solids (TSS), followed by physical filtration, flocculation, aerobic digestion, and clarification before discharging into the surface water [36]. These processes are expected to remove organic compounds through degradation, but the removal of soluble, inorganic elements is less effective [36].

In commercial waste water treatment plants, Na_2_SO_4_ is added to first remove salts and metals as a solid precipitate prior to being treated by other processes [36]. The solids generated are then dried and hauled to residual waste landfills. While the commercial processes are expected to precipitate dissolved cations and filter solid elements, they are not expected to impact dissolved anions, such as chlorides as well as total dissolved solids (TDS) [36]. Limited research data have shown that prior to the voluntarily cessation of sending hydraulic fracturing waste to wastewater treatment plants, high levels of barium (Ba), strontium (Sr), bromides (Br), chlorides (Cl), TDS, and benzene were detected from the effluent discharge not only from POTW but also from CWT [36]. For instance, in Josephine Brine Treatment Inc., a commercially operated industrial wastewater treatment plant, Ba was detected from the effluent discharge with a mean concentration of 27.3 mg/L, 14 times EPA's maximum concentration limit (MCL) in drinking water and 4 times the derived drinking water minimal risk level (MRL) for intermediate and chronic exposures for adult men; 4.7 times the derived drinking water MRL for intermediate and chronic exposures for adult women; and 9 times the derived drinking water MRL for intermediate and chronic exposures for children [38]. The concentration of Ba in the effluent is also 1.3 and 6.7 times EPA criteria maximum concentration (CMC, 21 mg/L) and continuous concentration (CCC, 4.1 mg/L), respectively, the criteria set to protect aquatic health [38].

The mean concentration of Sr detected in the effluent of same facility was 2,980 mg/L, more than 740 times of EPA's recommended level in finished municipal drinking water of 4 mg/L, 43, 51 and 97 times the derived Sr drinking water MRL's for intermediate exposures for adult men, adult women, and children, respectively [38]. It is worth noting that wastewater treatment facility in Pennsylvania is required to report to PADEP for routine discharge of a toxic with a concentration above 100 *μ*g/L (500 *μ*g/L for nonroutine discharge); however, the review of documents reveals no evidence of notification by waste treatment to PADEP [38]. No criteria have been established by EPA for the concentration of Br in the drinking water to minimize forming of halogenated byproducts, the presence of which in high concentrations may be linked to the increase in cancerous diseases [39]. In the effluent of the Josephine Brine Treatment plant, a mean concentration of 1,070 mg/L for Br was detected, 10,700 times the concentration of 0.1 mg/L reported to be associated with adverse health concerns [39]. Again, as for Sr, a careful review of the archived documents revealed no notification of the discharge of high concentrations of bromine to PADEP [39].

The concentration of above mentioned analytes in the effluent of wastewater treatment facilities decreased significantly after the discharge of hydraulic fracturing wastewater into surface water was discontinued per PADEP's request, indicating the elevated concentrations of inorganic analytes found in the effluent prior to the voluntarily cease of discharge were largely attributable to the fracturing fluids [36], although more extensive investigations are still needed to confirm [36]. In 2011, EPA in conjunction with PADEP further requested that when considering the acceptance of fracking wastewater, the wastewater treatment plants need to document the chemicals used in hydraulic fracturing process that could reasonably be expected to be present in the wastewater and assess their potential impact on wastewater treatment and the receiving waters [40, 41]. Since then, the tighter regulation influenced several wastewater treatment plants to stop or reduce receiving unconventional natural gas fracturing fluid [36].

Fontenot et al. [42] reported elevated concentrations of arsenic, selenium, strontium, and TDS in some private water wells located near an active natural gas extraction site in Barnett Shale formation; however, it is important to recognize that there were also a number of private water wells in close proximity to natural gas wells that showed no elevated constituents. The spatial and temporal geochemical signatures of brines collected from shallow aquifers are compared with that from deeper shale formations in the Appalachian basin in the northeastern portion of Pennsylvania to assess the migration possibility of hydraulic fracturing fluid [43]. The elevated Br/Cl ratio (>0.001) and low Na/Cl ratio (<5) among other geochemical signatures found in a subset of shallow aquifers samples that lack geospatial association with the nearest shale gas wells were not distinguishable from the samples collected historically from the deeper Appalachian formations in the 1980s, the time period that no gas drilling activities were ongoing in the region [43]. These data delineate the possible natural mixing between the Appalachian brines and shallow groundwater through natural flow paths (i.e., fracture zones) that occurs over time and refute the overly broad claim that hydraulic fracturing accounts for the elevated groundwater salinity in all locations. On the other hand, these data suggest that great caution should therefore be taken prior to granting hydraulic fracturing or wastewater disposal injection activities in these areas because of the preexisting network of cross-formational pathways that connect to shallow groundwater specifically under high hydrodynamic pressure [43].

The organic constituents of produced water (i.e., organic acids and semivolatile organics) have also been studied [37]. These organic constituents in produced water are either attributable to the fracturing fluid additives or are from the release of natural organic compounds associated with formation which could comprise a significant portion of the organic matrix of the produced water. Benzene, toluene, ethylene, and xylene (aka BTEX) are commonly found in produced water [44]. Diesel fuel which introduces BTEX has been used as an additive to increase the efficiency in transporting proppants in the fracturing fluids [16]. BTEX are relatively mobile as well as toxic and/or confirmed carcinogens [45]. In 2004, EPA entered into a Memorandum of Agreement (MOA) with major service companies to voluntarily eliminate diesel fuel from hydraulic fracturing fluids that are injected directly into underground sources of drinking water (USDWs) for coal-bed methane (coal seam gas) production [16]. Similarly, the identification of trace amount of BTEX in well water near Miles, in western Queensland, Australia, led to the ban of the use of BTEX chemicals in fracturing fluids with the concern of adverse impacts on ground water [46]. While the ban or phase out of the use of diesel fuel in the hydraulic fracturing will reduce the introduction of BTEX into groundwater, BTEX also naturally exists in the gas and coal-bed deposition, the hydraulic fracturing process itself can therefore release significant amounts of BTEX into produced water even without using diesel fuel. It has been reported that a significant portion of wells drilled nationwide (56%) produced both oil and natural gas [47]. This is consistent with the report by Sirivedhin and associates that revealed that benzene-based compounds, particularly BTEX, were the dominant organic species found in produced water samples generated from oil/coalbed methane wells [37]. In addition to BTEX, dissolved organic acids from the formation such as monocarboxylic and dicarboxylic acid anions comprise the bulk of dissolved organic species found in produced water.

The remaining portion of fracturing fluid that is left far beneath groundwater levels may induce the opening of long fissures over time which will allow fracturing fluid or natural gas to travel upward to reach groundwater and thus reducing its quality. It has been assumed that in Marcellus shale, hydraulic fractures are confined vertically and the hydraulic fracturing process is conducted thousands of feet below the deepest aquifers suitable for drinking water [19]. Recent findings might shift this paradigm. Osborn and colleagues demonstrate that in active gas-extraction areas with one or more gas wells within 1 km (3,280 feet) apart, both average and maximum methane concentrations in drinking-water wells increase with proximity to the nearest gas well (19.2 and 64 mg/L, resp.) in northeastern Pennsylvania [48]; in contrast, in neighboring nonextraction sites within similar geologic formations methane concentrations only averaged 1.1 mg/L and the farther away from natural gas development, the lower combustible gas concentrations were found in water wells [16, 23]. However, the study surveyed a relative small number of nonrandomized wells, of which several of the contaminated water wells reported in the study were from a region that had aquifer contamination in the past that might be associated with casing leaks or inadequate cementing of orphaned gas wells rather than hydraulic fracturing. In addition, Osborn's study did not include baseline measurements of levels of methane in aquifers prior to fracturing [49–51], without of which any definitive conclusions are questionable. Therefore, caution is needed to interpret and extrapolate Osborn's results systematically and long-term, coordinated sampling and monitoring procedures are required for future studies.

### 2.3. Wastewater Management Concerns

Public concern has been voiced about the potential leakage of fracturing wastewater into other water bodies, if not probably stored, treated, or disposed. Gross and associates have demonstrated that surface spills or leakage into the shallow water formations is a critical event and could account for most water quality issues associated with hydraulic fracturing [52]. Chemicals can potentially leach into groundwater through failures in the lining of ponds or containment systems most of which are constructed near the well sites to temporarily hold flowback/produced water [52–54]. Between 2009 and 2010, of the 4,000 permitted oil and natural gas wells in Marcellus Shale in Pennsylvania, there were 630 reported environmental health and safety violations of which half were associated with leaks and spills of the flowback/produced fluids [55]. In Weld County, Colorado, groundwater is the main source of water supply for local and commercial use. Some locations of Weld County have very shallow depth to water table with higher opportunities for groundwater contamination, yet Weld County has the highest density of wells used by hydraulic fracturing for both natural gas and crude oil in the United States. In Weld County, tank battery systems (for storing produced water and crude oil in various stages of separation) and production facilities (sources of hydrocarbons in the refining process) were found at most well sites which could contribute to leaks and spills [52]. BTEX for example, can pass through soil into the groundwater after spills. Gross assessed the profiles of BTEX concentrations over time by repeated sampling of groundwater on/near multiple spill sites either prior to or shortly after remediation began. In total, 90% of groundwater samples collected contained benzene and 30% of samples contained toluene concentrations above their MCLs. Although there was a delay between the reported surface spill date and the first water sampling date (BTEX is volatile; it is possible that the initial BTEX concentration shortly after spills could be even higher), nevertheless the mean concentration of benzene and toluene from groundwater samples collected from inside the excavation sites prior to the remediation was 280 and 2.2 times that of respective MCLs [52]. While these data indicate that benzene and toluene are of greater concern when considering BTEX groundwater concentrations from surface spills, the results in the report should be interpreted with caution. The distribution of BTEX concentrations collected at spill sites is highly skewed with the median values much lower than the estimated means, in some cases several hundredfold lower. For example, the median value for toluene does not exceed its respective MCL [52]. In addition, the study fails to differentiate the contamination sources of BTEX which could come from onsite oil spills, natural gas-related produced water spills, or a combination of the two, although produced water from gas production could have higher contents of BTEX than water from oil production [44].

Flowback/produced water reuse for hydraulic fracturing is another option chosen by an increasing number of oil and gas companies as it reduces wastewater generated, fresh water required, and wastewater management costs. Currently, most of the flowback water from Marcellus Shale in Pennsylvania is recycled and reused in future hydraulic fracturing processes, while industrial treatment and discharge into surface water declined to only 3% [56]. The fracturing fluid reuse option however is not without limitations. Not all produced water from the hydraulic fracturing is suitable for reuse. The chemical signatures as well as the concentrations of TDS, TSS, and brines in produced water largely depend on the nature of the formation [33]. Highly soluble TDS could be difficult and expensive to remove from wastewater, and their combination with various other contaminants necessitates multiple treatment technologies in sequence [56]. For instance, the quality of produced water from Haynesville Shale is considered less attractive for reuse potentials as it contains high levels of TDS, chlorides, and TSS and has high scaling tendency (high calcium and high magnesium). In contrast, produced water from Fayetteville Shale is considered to have excellent potential for reuse due to low concentrations of chlorides, TDS, and low scaling tendency [33]. In Marcellus Shale, however, only TSS, but not the salts in produced water, is filtered prior to reuse. Therefore, the concentrations of the remaining components (i.e., TDS/brines, scaling components) if beyond accepted range need to be diluted with substantial amounts of fresh water before the filtered produced water can be directly reused [33]. The amount of energy required to treat the fluids, the amount of air pollution generated, the amount of solid waste that will be disposed of in landfills, and the cost of logistics are among other factors that will determine the feasibility of the produced water reuse option. In addition, some shale formations tend to either “trap” or are considered as highly desiccated which render significant insufficient amounts of flowback fluid back to the surface [33].

Several other mechanisms for wastewater management have also been applied. Wastewater can be injected underground into Class II Wells [57, 58]. Class II disposal wells which account for approximately 20% of 144,000 Class II Wells in the United States can only be used to dispose fluids associated with oil and gas production. While UIC requirements do not apply to hydraulic fracturing drilling operation (with exception when diesel fuel is used), the underground injection of wastewater generated during oil and gas production (including hydraulic fracturing) requires an Underground Injection (UIC) permit under the SDWA [58]. In many regions of the United States, underground injection is the most common method of disposing fluids or other substances generated from shale gas extraction operations [59]. For instance, approximately 98% of all brine is disposed of by injection back into brine-bearing or depleted oil and gas formations deep below the earth's surface in Ohio [60].

While wastewater injection into Class II Wells may be an ideal solution for some states perhaps due to the presence of suitable geological formation in the area as well as the availability of sufficient wells, this option may be problematic for other states that have already reached their maximum injection capacity or are unable to use this option due to unsuitable geology [43, 61]. The existence of cross-formational pathways allowing deep saline water to migrate upward into shallow, fresher aquifers has been documented in Appalachian Basin as well as in other areas across the nation [43]. Furthermore, in Pennsylvania, more than 180,000 wells had been drilled prior to any requirement for documenting the locations. The location of many wells is unknown, while others have been improperly abandoned [62]. The existence of these wells might increase the chance of injected waste fluids escaping the injection formation through the connecting fractures and transport to higher aquifer, although this issue is still up for debate [63, 64]. As a result, wastewater in Pennsylvania has been transported to nearby states for treatment which presents concerns of increased possibility of leaks or spills during transportation [32]. Clearly, more data are required in order to further evaluate the movement of contaminations along pathways either from wellbores or from deep formations to overlying groundwater.

The wastewater can also be treated and reused in irrigation, for unpaved road dust control, or even roads deicing, although the portion of the waster fluid used for these purposes is very small, having received a high degree of criticism and is discouraged [35, 56, 65]. In these cases, the wastewater is either treated or mixed with large volumes of fresh water to lower its TDS and other constituents to acceptable ranges [33]. These solutions inevitably require additional water withdrawals, increased onsite storage capacity, increased cost of transportation, and requires more resources and chemicals for treatment purposes. Moreover, the use of wastewater for crop irrigation or unpaved road dust control may pose additional health threat due to the unknown toxicity of many individual components attributable to the trade secret nature of many constitutes used by hydraulic fracturing companies [65]. While no published data are currently available for assessing the potential environmental and human and animal health impacts of treated produced water used for abovementioned purposes, we have learned a very expensive lesson in the Times Beach site in Missouri at the expense of the disincorporation of the city [66]. The site was sprayed with waste oil in early 1970s for unpaved dust control; in 1982, EPA later found the oil to be contaminated with dioxins which are highly toxic and can cause severe reproductive and developmental problems.

## 3. Conclusion

While hydraulic fracturing may present an economic advantage to the United States by transitioning the country to an energy independent state, there are several environmental concerns associated with the process that have not been properly addressed.

Primarily, a main concern of the public and environmentalists pertaining to hydraulic fracturing is governmental leniency in its regulation. In the 2005 Energy Policy Act, Congress revised the SDWA definition of “underground injection” to specifically exclude the “underground injection of fluids or propping agents (other than diesel fuels) pursuant to hydraulic fracturing operations related to oil, gas, or geothermal production activities” from UIC regulation (SDWA Section 1421(d)(1)(B)) [67]. Between 2005 and 2009, the 14 leading oil and gas service companies used 780 million gallons of chemical products in fracturing fluids [68]. The concentration and composition of the fluid used in hydraulic fracturing vary with the nature of the formation. Although some of these chemicals may be harmless, others are not well investigated and may be hazardous to human health and the environment. To allow for a competitive market in the field of hydraulic fracturing, under current regulation, oil and gas companies are not required to disclose the identity of the chemicals in their fracturing fluids other than under the Emergency Planning and Community Right-to-Know Act (EPCRA), under which owners or operators of facilities where certain hazardous hydraulic fracturing chemicals are present above certain thresholds may have to comply with emergency planning requirements, emergency release notification obligations, and hazardous chemical storage reporting requirements [69]. While disposal management of fracturing wastes is regulated, Provisions of the Resource Conservation and Recovery Act (RCRA) exempt drilling fluids, produced water, and other wastes associated with the exploration, development, or production of crude oil, natural gas, or geothermal energy from regulation as hazardous wastes under Subtitle C of RCRA. As a consequence, instead of being disposed into Class I Wells which are designated for both hazardous and nonhazardous waste, wastewater is injected into Class II Wells which are far less regulated than Class I [69]. In addition, appropriate documentation/registration/reporting systems for hydraulic fracturing related activities are loosely enforced and the implementation status of current law regulation is less than satisfactory. In August 2013, the United States Government Accountability Office (GAO) pointed out that current Bureau of Land Management's (BLM) environmental inspection prioritization process may miss oil and gas wells that could pose the greatest environmental risk [70]. BLM manages onshore federal oil and gas resources and ensure that oil and gas operations on federal lands are prudently conducted in a manner that ensures protection of the surface and subsurface environment. However, approximately 41% of the 60,330 federal oil and gas wells including those used for wastewater disposal found no record of ever having received an environmental inspection between 2007 and 2012 [70]. Similarly, in the case of hydraulic fracturing fluid spills, the majority of reports did not include information specifying the volume of produced water that was spilled [52].

Hydraulic fracturing is a major investment of several countries globally. Canada, South Africa, Germany, United Kingdom, Russia, and China all use fracturing techniques to increase their natural gas production. The choice to continuously conduct hydraulic fracturing is currently under debate in the United Kingdom due to public concerns of its potential environmental impacts [71]. France and Bulgaria on the other hand have banned the use of hydraulic fracturing for gas extraction because of environmental concerns [72, 73]. In United States, if hydraulic fracturing results in the release of hazardous substances at or under the surface in a manner that may endanger public health or the environment [69], all potentially responsible parties could face liability under CERCLA for cleanup costs, natural resource damages, and the costs of federal public health studies. However, federal regulations on fracturing overall have not been stringent enough. Proper documentation/reporting systems for wastewater discharge and spills need to be enforced at the federal, state, and industrial level and UIC requirements under SDWA should be extended to hydraulic fracturing operations regardless if diesel fuel is used as a fracturing fluid (or a component of a fracturing fluid) or not. Furthermore, federal laws mandating hydraulic companies to disclose fracturing fluid composition and concentration not only to federal and state regulatory agencies but also to health care professionals would encourage this practice. Only the full disclosure of fracturing chemicals will allow future research to fill the knowledge gaps for a better understanding of the impacts of hydraulic fracturing on human health and environment [19, 20] as well as to determine if any further regulations or the improvement of technology itself are needed.

## Figures and Tables

**Table 1:** Chemical disclosure requirements by states.

Chemical disclosure required to the state		No chemical disclosure required to the state
Disclosure of trade secret to medical personnel	No disclosure of trade secret to medical personnel	
Arkansas	Alabama	Alaska, California
Colorado*	Indiana	Illinois, Kansas
Montana*	Louisiana	Kentucky, Mississippi
Ohio	Michigan	Missouri, Nebraska
Pennsylvania*	New Mexico	New York, South Dakota
Texas*	North Dakota	Tennessee, Utah
	Oklahoma	Virginia, Washington
	West Virginia	
	Wyoming	

*States requiring physicians to sign confidentiality agreements.
